# (μ-2-Pyridine­aldazine-κ^4^ *N*,*N*′:*N*′′,*N*′′′)bis­[bis­(*N*,*N*-di-*n*-propyl­dithio­carbamato-κ^2^ *S*,*S*′)cadmium(II)]

**DOI:** 10.1107/S1600536808025889

**Published:** 2008-08-16

**Authors:** Pavel Poplaukhin, Edward R. T. Tiekink

**Affiliations:** a Department of Chemistry, University of Texas at San Antonio, One UTSA Circle, San Antonio, TX 78249-0698, USA

## Abstract

The dinuclear centrosymmetric title compound, [Cd_2_(C_7_H_14_NS_2_)_4_(C_12_H_10_N_4_)], features a tetra­dentate 2-pyridine­aldazine ligand that chelates two Cd centres. The coordination geometry for Cd is distorted octa­hedral based on a *cis*-N_2_S_4_ donor set. In the crystal structure, mol­ecules are connected into a supra­molecular chain aligned along the *a* direction *via* C—H⋯S and C—H⋯π contacts, and by π–π contacts [centroid-to-centroid distance 3.5708 (15) Å]. The *n*-propyl groups are each disordered, one equally over two sites and the other with a site-occupancy factor of 0.618 (8) for the major component.

## Related literature

For background literature, see: Tiekink (2006 ▶); Benson *et al.* (2007 ▶). For a related structure, see: Lai & Tiekink (2006 ▶).
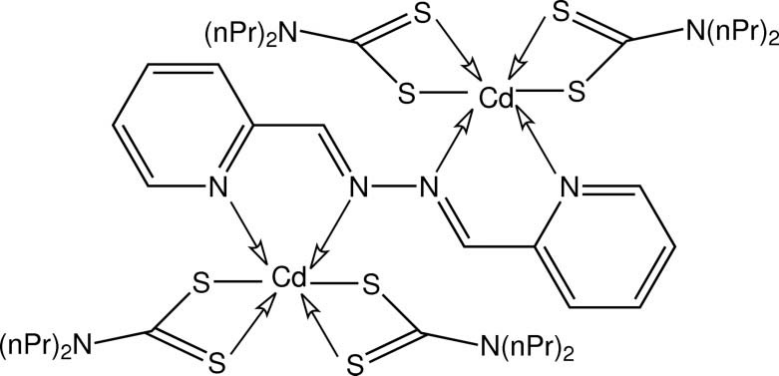


## Experimental

### 

#### Crystal data

[Cd_2_(C_7_H_14_NS_2_)_4_(C_12_H_10_N_4_)]
*M* *_r_* = 1140.39
Monoclinic, 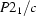

*a* = 9.0768 (16) Å
*b* = 11.137 (2) Å
*c* = 25.389 (5) Å
β = 92.216 (3)°
*V* = 2564.7 (8) Å^3^
*Z* = 2
Mo *K*α radiation
μ = 1.19 mm^−1^
*T* = 98 (2) K
0.35 × 0.12 × 0.10 mm

#### Data collection

Rigaku AFC12κ/SATURN724 diffractometer
Absorption correction: multi-scan (*ABSCOR*; Higashi, 1995 ▶) *T*_min_ = 0.656, *T*_max_ = 1 (expected range = 0.582–0.888)
20914 measured reflections
5861 independent reflections
5539 reflections with *I* > 2σ(*I*)
*R*_int_ = 0.031

#### Refinement

*R*[*F* ^2^ > 2σ(*F* ^2^)] = 0.031
*wR*(*F* ^2^) = 0.077
*S* = 1.08
5861 reflections
299 parameters
H-atom parameters constrained
Δρ_max_ = 0.71 e Å^−3^
Δρ_min_ = −0.72 e Å^−3^

### 

Data collection: *CrystalClear* (Rigaku/MSC, 2005 ▶); cell refinement: *CrystalClear*; data reduction: *CrystalClear*; program(s) used to solve structure: *SIR92* (Altomare *et al.*, 1994 ▶); program(s) used to refine structure: *SHELXL97* (Sheldrick, 2008 ▶); molecular graphics: *ORTEPII* (Johnson, 1976 ▶) and *DIAMOND* (Brandenburg, 2006 ▶); software used to prepare material for publication: *SHELXL97*.

## Supplementary Material

Crystal structure: contains datablocks global, I. DOI: 10.1107/S1600536808025889/ng2485sup1.cif

Structure factors: contains datablocks I. DOI: 10.1107/S1600536808025889/ng2485Isup2.hkl

Additional supplementary materials:  crystallographic information; 3D view; checkCIF report

## Figures and Tables

**Table 1:** Hydrogen-bond geometry (Å, °) *Cg* is the centroid of the N3/C15–C19 ring.

*D*—H⋯*A*	*D*—H	H⋯*A*	*D*⋯*A*	*D*—H⋯*A*
C9—H9*B*⋯S4^i^	0.99	2.83	3.815 (3)	171
C16—H16⋯S3^ii^	0.95	2.82	3.674 (3)	150
C3—H3*B*⋯*Cg*^iii^	0.95	2.99	3.853 (5)	147

Symmetry codes: (i) 
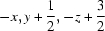
; (ii) 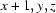; (iii) 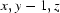.

**Fig. 1.**
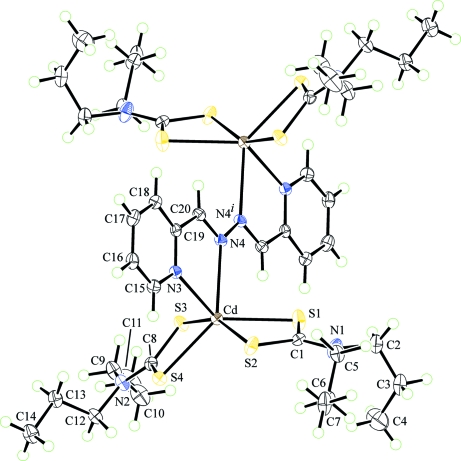
Molecular structure of (I) showing the crystallographic numbering scheme. Displacement ellipsoids are shown at the 50% probability level. Unlabelled atoms are related by the symmetry operation i: -x, 1 - y, 1 - z. The minor components of the disordered n-propyl groups omitted for clarity.

**Fig. 2.**
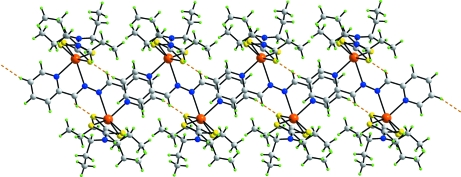
View of a supramolecular chain in (I) highlighting the overlap of the aromatic rings to allow the formation of the π–π interactions. The C—H···S3 interactions are shown as orange dashed lines. Colour code: Cd (orange), S (yellow), O (red), N (blue), C (grey) & H (green).

**Fig. 3.**
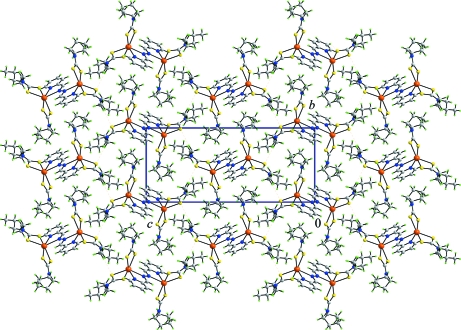
View in projection of the crystal packing in (I). Colour code as for Fig. 2.

## supplementary crystallographic information

### Comment

Metal dithiocarbamates have recently been applied in crystal engineering studies (*e.g.* Tiekink, 2006; Benson *et al.*, 2007). The title compound, {[(nPr)_2_NCS_2_]_2_Cd(2-C_5_H_4_N—C(H)═N—N═C(H)C_5_H_4_N-2)- Cd[S_2_CN(nPr)_2_]_2_} (I), features a centrosymmetric tetradentate 2-pyridinealdazine ligand coordinating to two Cd centres each of which is chelated by two dithiocarbamate ligands, Fig. 1. The molecule is centrosymmetric about the central N—N bond. The range of Cd—S bond distances is relatively narrow at 2.6124 (8) to 2.7165 (7) Å, but the Cd—N bond formed by the pyridine-N of 2.377 (2) Å is significantly shorter than the Cd—N bond distance of 2.6211 (19) Å formed with the azo-N atom. The coordination geometry is based on an octahedron within a *cis*-N_2_S_4_ donor set. The structure reported here for (I) resembles closely the dithiophosphate analogue (Lai & Tiekink, 2006). In the crystal structure, molecules are connected into a supramolecular chain, aligned along the a-direction and illustrated in Fig. 2, *via* C—H···S3 and π–π contacts. The latter occur between centrosymmetically related N3,C15—C19 rings with *Cg*···*Cg* = 3.5708 (15) Å. These are consolidated into the crystal structure *via* additional C—H···S4 contacts and C—H···π interactions, Table 1 and Fig. 3.

### Experimental

Compound (I) was prepared by standard methods (Benson *et al.*, 2007) and red crystals were grown by the slow evaporation of a methanol–ethanol (1/1) solution of (I), m.p. 441-443 K. IR (cm^-1^): 1474 (s, C═N), 1171 (s, C—S). TGA: One broad step with onset = 625 K, midpoint = 662 K, and endset = 711 K which corresponds to decomposition leading to CdS (mass loss 79.4% *cf.* theoretical mass loss = 74.4%).

### Refinement

The H atoms were geometrically placed (C—H = 0.95–0.99 Å) and refined as riding with *U*_iso_(H) = 1.2*U*_eq_(C) or 1.5*U*_eq_(methyl-C). The N1-bound n-propyl groups were each found to be disordered. The C6—C7 residue was disordered over two positions, each with 50% site occupancy factors (from anisotropic refinement). The C3 atom of the C2—C4 residue was disordered over two positions with the major component having a site occupancy factor = 0.618 (8) (from anisotropic refinement).

### Crystal data

**Table:**

[Cd_2_(C_7_H_14_NS_2_)_4_(C_12_H_10_N_4_)]	*F*(000) = 1172
*M**_r_* = 1140.39	*D*_x_ = 1.477 Mg m^−^^3^
Monoclinic, *P*2_1_/*c*	Mo *K*α radiation, λ = 0.71070 Å
Hall symbol: -P 2ybc	Cell parameters from 16020 reflections
*a* = 9.0768 (16) Å	θ = 1.8–40.5°
*b* = 11.137 (2) Å	µ = 1.19 mm^−^^1^
*c* = 25.389 (5) Å	*T* = 98 K
β = 92.216 (3)°	Prism, red
*V* = 2564.7 (8) Å^3^	0.35 × 0.12 × 0.10 mm
*Z* = 2	

### Data collection

**Table:**

Rigaku AFC12κ/SATURN724 diffractometer	5861 independent reflections
Radiation source: fine-focus sealed tube	5539 reflections with *I* > 2σ(*I*)
graphite	*R*_int_ = 0.031
ω scans	θ_max_ = 27.5°, θ_min_ = 2.3°
Absorption correction: multi-scan (*ABSCOR*; Higashi, 1995)	*h* = −11→11
*T*_min_ = 0.656, *T*_max_ = 1	*k* = −14→14
20914 measured reflections	*l* = −32→31

### Refinement

**Table:**

Refinement on *F*^2^	Primary atom site location: structure-invariant direct methods
Least-squares matrix: full	Secondary atom site location: difference Fourier map
*R*[*F*^2^ > 2σ(*F*^2^)] = 0.031	Hydrogen site location: inferred from neighbouring sites
*wR*(*F*^2^) = 0.077	H-atom parameters constrained
*S* = 1.08	*w* = 1/[σ^2^(*F*_o_^2^) + (0.0362*P*)^2^ + 2.6654*P*] where *P* = (*F*_o_^2^ + 2*F*_c_^2^)/3
5861 reflections	(Δ/σ)_max_ = 0.001
299 parameters	Δρ_max_ = 0.71 e Å^−^^3^
0 restraints	Δρ_min_ = −0.72 e Å^−^^3^

### Special details

**Table:**

Geometry. All e.s.d.'s (except the e.s.d. in the dihedral angle between two l.s. planes) are estimated using the full covariance matrix. The cell e.s.d.'s are taken into account individually in the estimation of e.s.d.'s in distances, angles and torsion angles; correlations between e.s.d.'s in cell parameters are only used when they are defined by crystal symmetry. An approximate (isotropic) treatment of cell e.s.d.'s is used for estimating e.s.d.'s involving l.s. planes.
Refinement. Refinement of *F*^2^ against ALL reflections. The weighted *R*-factor *wR* and goodness of fit *S* are based on *F*^2^, conventional *R*-factors *R* are based on *F*, with *F* set to zero for negative *F*^2^. The threshold expression of *F*^2^ > σ(*F*^2^) is used only for calculating *R*-factors(gt) *etc*. and is not relevant to the choice of reflections for refinement. *R*-factors based on *F*^2^ are statistically about twice as large as those based on *F*, and *R*- factors based on ALL data will be even larger.

### Fractional atomic coordinates and isotropic or equivalent isotropic displacement parameters (Å^2^)

**Table:**

	*x*	*y*	*z*	*U*_iso_*/*U*_eq_	Occ. (<1)
Cd	0.153452 (17)	0.407355 (14)	0.605114 (6)	0.01585 (6)	
S1	0.07349 (7)	0.19908 (6)	0.56627 (3)	0.03080 (16)	
S2	0.37230 (6)	0.24128 (5)	0.61348 (3)	0.02499 (14)	
S3	−0.06899 (6)	0.53972 (6)	0.63409 (2)	0.02094 (12)	
S4	0.19579 (6)	0.48599 (5)	0.70265 (2)	0.01910 (12)	
N2	−0.0279 (2)	0.6233 (2)	0.73187 (8)	0.0204 (4)	
N3	0.3282 (2)	0.53991 (17)	0.56901 (8)	0.0170 (4)	
N4	0.06743 (19)	0.50248 (18)	0.51464 (7)	0.0162 (4)	
C1	0.2466 (2)	0.1454 (2)	0.58446 (9)	0.0181 (4)	
C2	0.1771 (3)	−0.0570 (2)	0.55108 (11)	0.0278 (5)	0.618 (8)
H2A	0.2185	−0.0870	0.5181	0.033*	0.618 (8)
H2B	0.0832	−0.0156	0.5417	0.033*	0.618 (8)
C3	0.1459 (5)	−0.1621 (4)	0.5862 (2)	0.0325 (13)	0.618 (8)
H3A	0.0719	−0.2141	0.5678	0.039*	0.618 (8)
H3B	0.2376	−0.2096	0.5912	0.039*	0.618 (8)
C4	0.0910 (4)	−0.1317 (4)	0.63923 (14)	0.0540 (10)	0.618 (8)
H4A	0.0742	−0.2057	0.6590	0.081*	0.618 (8)
H4B	−0.0017	−0.0869	0.6351	0.081*	0.618 (8)
H4C	0.1644	−0.0823	0.6585	0.081*	0.618 (8)
C80	0.1771 (3)	−0.0570 (2)	0.55108 (11)	0.0278 (5)	0.382 (8)
H80A	0.2327	−0.1285	0.5399	0.033*	0.382 (8)
H80B	0.1301	−0.0204	0.5191	0.033*	0.382 (8)
C30	0.0531 (8)	−0.0980 (6)	0.5891 (3)	0.031 (2)	0.382 (8)
H30A	−0.0184	−0.0312	0.5912	0.037*	0.382 (8)
H30B	0.0002	−0.1663	0.5720	0.037*	0.382 (8)
C40	0.0910 (4)	−0.1317 (4)	0.63923 (14)	0.0540 (10)	0.382 (8)
H40A	0.0021	−0.1544	0.6576	0.081*	0.382 (8)
H40B	0.1398	−0.0645	0.6578	0.081*	0.382 (8)
H40C	0.1584	−0.2003	0.6385	0.081*	0.382 (8)
N1	0.2807 (2)	0.0299 (2)	0.57585 (10)	0.0293 (5)	0.50
C5	0.4367 (6)	−0.0111 (5)	0.5768 (3)	0.0210 (11)	0.50
H5A	0.5005	0.0539	0.5641	0.025*	0.50
H5B	0.4462	−0.0812	0.5532	0.025*	0.50
C6	0.4858 (5)	−0.0460 (5)	0.6335 (2)	0.0235 (10)	0.50
H6A	0.4651	0.0209	0.6578	0.028*	0.50
H6B	0.4298	−0.1173	0.6447	0.028*	0.50
C7	0.6503 (6)	−0.0740 (5)	0.6360 (3)	0.0325 (13)	0.50
H7A	0.6809	−0.0963	0.6721	0.049*	0.50
H7B	0.7054	−0.0029	0.6254	0.049*	0.50
H7C	0.6702	−0.1407	0.6122	0.049*	0.50
N10	0.2807 (2)	0.0299 (2)	0.57585 (10)	0.0293 (5)	0.50
C50	0.4217 (7)	−0.0203 (5)	0.6042 (3)	0.0279 (13)	0.50
H50A	0.4466	0.0270	0.6363	0.033*	0.50
H50B	0.4068	−0.1051	0.6144	0.033*	0.50
C60	0.5433 (7)	−0.0108 (5)	0.5656 (3)	0.0360 (13)	0.50
H60A	0.5545	0.0740	0.5547	0.043*	0.50
H60B	0.5169	−0.0586	0.5337	0.043*	0.50
C70	0.6906 (7)	−0.0567 (6)	0.5903 (3)	0.0447 (16)	0.50
H70A	0.7677	−0.0504	0.5645	0.067*	0.50
H70B	0.6797	−0.1408	0.6009	0.067*	0.50
H70C	0.7180	−0.0081	0.6214	0.067*	0.50
C8	0.0275 (2)	0.5569 (2)	0.69392 (9)	0.0178 (4)	
C9	−0.1698 (3)	0.6864 (2)	0.72549 (11)	0.0263 (5)	
H9A	−0.1865	0.7086	0.6880	0.032*	
H9B	−0.1652	0.7615	0.7464	0.032*	
C10	−0.2992 (3)	0.6109 (3)	0.74297 (12)	0.0355 (7)	
H10A	−0.2835	0.5887	0.7805	0.043*	
H10B	−0.3050	0.5360	0.7220	0.043*	
C11	−0.4437 (3)	0.6802 (4)	0.73570 (13)	0.0472 (9)	
H11A	−0.5253	0.6304	0.7473	0.071*	
H11B	−0.4602	0.7007	0.6984	0.071*	
H11C	−0.4383	0.7540	0.7567	0.071*	
C12	0.0537 (3)	0.6428 (2)	0.78277 (9)	0.0234 (5)	
H12A	0.1123	0.5701	0.7918	0.028*	
H12B	−0.0177	0.6551	0.8108	0.028*	
C13	0.1564 (3)	0.7511 (2)	0.78105 (10)	0.0242 (5)	
H13A	0.0972	0.8253	0.7767	0.029*	
H13B	0.2198	0.7439	0.7503	0.029*	
C14	0.2533 (3)	0.7595 (3)	0.83168 (11)	0.0304 (6)	
H14A	0.3183	0.8295	0.8297	0.046*	
H14B	0.3131	0.6865	0.8357	0.046*	
H14C	0.1906	0.7678	0.8620	0.046*	
C15	0.4586 (3)	0.5575 (2)	0.59424 (10)	0.0202 (5)	
H15	0.4744	0.5230	0.6282	0.024*	
C16	0.5719 (3)	0.6238 (2)	0.57324 (10)	0.0215 (5)	
H16	0.6628	0.6342	0.5925	0.026*	
C17	0.5501 (3)	0.6745 (2)	0.52364 (10)	0.0227 (5)	
H17	0.6262	0.7192	0.5080	0.027*	
C18	0.4137 (3)	0.6584 (2)	0.49713 (9)	0.0193 (4)	
H18	0.3948	0.6930	0.4633	0.023*	
C19	0.3063 (2)	0.59112 (19)	0.52103 (9)	0.0161 (4)	
C20	0.1614 (2)	0.5730 (2)	0.49415 (9)	0.0169 (4)	
H20	0.1376	0.6131	0.4619	0.020*	

### Atomic displacement parameters (Å^2^)

**Table:**

	*U*^11^	*U*^22^	*U*^33^	*U*^12^	*U*^13^	*U*^23^
Cd	0.01450 (9)	0.01688 (9)	0.01615 (10)	0.00097 (6)	0.00017 (6)	−0.00067 (6)
S1	0.0254 (3)	0.0220 (3)	0.0437 (4)	0.0073 (2)	−0.0170 (3)	−0.0097 (3)
S2	0.0160 (3)	0.0172 (3)	0.0413 (4)	−0.0007 (2)	−0.0038 (2)	0.0019 (2)
S3	0.0162 (3)	0.0263 (3)	0.0201 (3)	0.0040 (2)	−0.0029 (2)	−0.0056 (2)
S4	0.0159 (3)	0.0221 (3)	0.0192 (3)	0.0031 (2)	−0.0004 (2)	−0.0007 (2)
N2	0.0146 (9)	0.0258 (10)	0.0207 (10)	0.0011 (8)	0.0002 (7)	−0.0066 (8)
N3	0.0169 (9)	0.0166 (9)	0.0172 (9)	0.0019 (7)	−0.0018 (7)	−0.0009 (7)
N4	0.0135 (9)	0.0192 (9)	0.0156 (9)	−0.0008 (7)	−0.0038 (7)	−0.0006 (7)
C1	0.0185 (10)	0.0167 (10)	0.0193 (11)	0.0003 (8)	0.0025 (8)	0.0035 (9)
C2	0.0275 (13)	0.0207 (12)	0.0348 (14)	−0.0004 (10)	−0.0021 (10)	−0.0054 (11)
C3	0.031 (2)	0.022 (2)	0.045 (3)	−0.0029 (18)	−0.0006 (19)	−0.0092 (19)
C4	0.046 (2)	0.075 (3)	0.0421 (19)	−0.0291 (19)	0.0068 (15)	−0.0073 (18)
C80	0.0275 (13)	0.0207 (12)	0.0348 (14)	−0.0004 (10)	−0.0021 (10)	−0.0054 (11)
C30	0.026 (4)	0.022 (4)	0.043 (4)	−0.006 (3)	−0.003 (3)	−0.005 (3)
C40	0.046 (2)	0.075 (3)	0.0421 (19)	−0.0291 (19)	0.0068 (15)	−0.0073 (18)
N1	0.0196 (10)	0.0189 (10)	0.0493 (14)	0.0004 (8)	−0.0019 (9)	−0.0003 (10)
C5	0.014 (3)	0.023 (3)	0.026 (3)	0.0064 (18)	0.008 (2)	−0.008 (3)
C6	0.013 (2)	0.022 (2)	0.034 (3)	0.0030 (18)	−0.011 (2)	0.005 (2)
C7	0.019 (2)	0.022 (2)	0.056 (4)	0.0074 (19)	−0.011 (2)	−0.012 (2)
N10	0.0196 (10)	0.0189 (10)	0.0493 (14)	0.0004 (8)	−0.0019 (9)	−0.0003 (10)
C50	0.029 (3)	0.020 (3)	0.035 (4)	0.004 (2)	0.004 (3)	−0.003 (3)
C60	0.037 (4)	0.027 (3)	0.044 (3)	0.007 (2)	−0.003 (3)	0.002 (2)
C70	0.028 (3)	0.037 (3)	0.070 (5)	0.006 (3)	−0.001 (3)	−0.006 (3)
C8	0.0148 (10)	0.0182 (10)	0.0206 (11)	−0.0026 (8)	0.0029 (8)	−0.0004 (9)
C9	0.0171 (11)	0.0322 (13)	0.0294 (13)	0.0073 (10)	0.0000 (9)	−0.0119 (11)
C10	0.0184 (12)	0.0529 (18)	0.0356 (15)	0.0001 (12)	0.0065 (10)	−0.0130 (14)
C11	0.0168 (13)	0.082 (3)	0.0430 (17)	0.0039 (14)	0.0017 (11)	−0.0247 (18)
C12	0.0218 (11)	0.0296 (13)	0.0188 (11)	0.0009 (10)	0.0001 (9)	−0.0060 (10)
C13	0.0192 (11)	0.0249 (12)	0.0279 (13)	0.0031 (9)	−0.0042 (9)	−0.0050 (10)
C14	0.0236 (12)	0.0339 (14)	0.0329 (14)	0.0053 (11)	−0.0082 (10)	−0.0069 (11)
C15	0.0184 (11)	0.0198 (11)	0.0221 (11)	0.0017 (9)	−0.0051 (8)	0.0007 (9)
C16	0.0154 (10)	0.0198 (11)	0.0288 (12)	0.0000 (9)	−0.0046 (9)	−0.0037 (10)
C17	0.0180 (11)	0.0181 (11)	0.0319 (13)	−0.0008 (9)	0.0013 (9)	−0.0005 (10)
C18	0.0191 (11)	0.0177 (11)	0.0212 (11)	−0.0008 (8)	0.0013 (8)	0.0001 (9)
C19	0.0169 (10)	0.0141 (10)	0.0173 (11)	0.0016 (8)	0.0007 (8)	−0.0030 (8)
C20	0.0164 (10)	0.0187 (10)	0.0156 (10)	0.0022 (8)	0.0002 (8)	−0.0017 (8)

### Geometric parameters (Å, °)

**Table:**

Cd—N3	2.377 (2)	C6—H6A	0.9900
Cd—S1	2.6124 (8)	C6—H6B	0.9900
Cd—N4	2.6211 (19)	C7—H7A	0.9800
Cd—S3	2.6279 (7)	C7—H7B	0.9800
Cd—S4	2.6407 (7)	C7—H7C	0.9800
Cd—S2	2.7165 (7)	N10—C50	1.548 (7)
S1—C1	1.727 (2)	C50—C60	1.508 (9)
S2—C1	1.709 (2)	C50—H50A	0.9900
S3—C8	1.735 (2)	C50—H50B	0.9900
S4—C8	1.726 (2)	C60—C70	1.543 (8)
N2—C8	1.329 (3)	C60—H60A	0.9900
N2—C9	1.470 (3)	C60—H60B	0.9900
N2—C12	1.480 (3)	C70—H70A	0.9800
N3—C15	1.338 (3)	C70—H70B	0.9800
N3—C19	1.353 (3)	C70—H70C	0.9800
N4—C20	1.284 (3)	C9—C10	1.525 (4)
N4—N4^i^	1.409 (3)	C9—H9A	0.9900
C1—N10	1.342 (3)	C9—H9B	0.9900
C1—N1	1.342 (3)	C10—C11	1.527 (4)
C2—N1	1.474 (3)	C10—H10A	0.9900
C2—C3	1.505 (5)	C10—H10B	0.9900
C2—H2A	0.9900	C11—H11A	0.9800
C2—H2B	0.9900	C11—H11B	0.9800
C3—C4	1.493 (6)	C11—H11C	0.9800
C3—H3A	0.9900	C12—C13	1.526 (4)
C3—H3B	0.9900	C12—H12A	0.9900
C4—H4A	0.9800	C12—H12B	0.9900
C4—H4B	0.9800	C13—C14	1.532 (3)
C4—H4C	0.9800	C13—H13A	0.9900
C80—N10	1.474 (3)	C13—H13B	0.9900
C80—C30	1.578 (8)	C14—H14A	0.9800
C80—H80A	0.9900	C14—H14B	0.9800
C80—H80B	0.9900	C14—H14C	0.9800
C30—C40	1.358 (9)	C15—C16	1.389 (3)
C30—H30A	0.9900	C15—H15	0.9500
C30—H30B	0.9900	C16—C17	1.387 (4)
C40—H40A	0.9800	C16—H16	0.9500
C40—H40B	0.9800	C17—C18	1.398 (3)
C40—H40C	0.9800	C17—H17	0.9500
N1—C5	1.487 (5)	C18—C19	1.388 (3)
C5—C6	1.539 (8)	C18—H18	0.9500
C5—H5A	0.9900	C19—C20	1.472 (3)
C5—H5B	0.9900	C20—H20	0.9500
C6—C7	1.524 (7)		
			
N3—Cd—S1	125.86 (5)	C6—C7—H7A	109.5
N3—Cd—N4	65.89 (6)	C6—C7—H7B	109.5
S1—Cd—N4	87.65 (5)	H7A—C7—H7B	109.5
N3—Cd—S3	106.92 (5)	C6—C7—H7C	109.5
S1—Cd—S3	113.49 (2)	H7A—C7—H7C	109.5
N4—Cd—S3	79.19 (4)	H7B—C7—H7C	109.5
N3—Cd—S4	94.63 (5)	C1—N10—C80	123.5 (2)
S1—Cd—S4	132.59 (2)	C1—N10—C50	117.5 (3)
N4—Cd—S4	135.68 (5)	C80—N10—C50	117.6 (3)
S3—Cd—S4	68.714 (18)	C60—C50—N10	106.6 (6)
N3—Cd—S2	87.49 (5)	C60—C50—H50A	110.4
S1—Cd—S2	67.45 (2)	N10—C50—H50A	110.4
N4—Cd—S2	122.40 (5)	C60—C50—H50B	110.4
S3—Cd—S2	158.05 (2)	N10—C50—H50B	110.4
S4—Cd—S2	94.19 (2)	H50A—C50—H50B	108.6
C1—S1—Cd	88.28 (8)	C50—C60—C70	110.9 (5)
C1—S2—Cd	85.28 (8)	C50—C60—H60A	109.5
C8—S3—Cd	86.54 (8)	C70—C60—H60A	109.5
C8—S4—Cd	86.30 (8)	C50—C60—H60B	109.5
C8—N2—C9	122.7 (2)	C70—C60—H60B	109.5
C8—N2—C12	121.6 (2)	H60A—C60—H60B	108.1
C9—N2—C12	115.64 (19)	C60—C70—H70A	109.5
C15—N3—C19	117.8 (2)	C60—C70—H70B	109.5
C15—N3—Cd	119.82 (16)	H70A—C70—H70B	109.5
C19—N3—Cd	122.14 (15)	C60—C70—H70C	109.5
C20—N4—N4^i^	112.8 (2)	H70A—C70—H70C	109.5
C20—N4—Cd	114.97 (14)	H70B—C70—H70C	109.5
N4^i^—N4—Cd	132.07 (19)	N2—C8—S4	121.29 (18)
N10—C1—N1	0.0 (2)	N2—C8—S3	120.26 (17)
N10—C1—S2	120.96 (18)	S4—C8—S3	118.45 (13)
N1—C1—S2	120.96 (18)	N2—C9—C10	112.7 (2)
N10—C1—S1	120.08 (18)	N2—C9—H9A	109.1
N1—C1—S1	120.08 (18)	C10—C9—H9A	109.1
S2—C1—S1	118.96 (14)	N2—C9—H9B	109.1
N1—C2—C3	112.9 (3)	C10—C9—H9B	109.1
N1—C2—H2A	109.0	H9A—C9—H9B	107.8
C3—C2—H2A	109.0	C9—C10—C11	110.7 (3)
N1—C2—H2B	109.0	C9—C10—H10A	109.5
C3—C2—H2B	109.0	C11—C10—H10A	109.5
H2A—C2—H2B	107.8	C9—C10—H10B	109.5
C4—C3—C2	115.8 (3)	C11—C10—H10B	109.5
C4—C3—H3A	108.3	H10A—C10—H10B	108.1
C2—C3—H3A	108.3	C10—C11—H11A	109.5
C4—C3—H3B	108.3	C10—C11—H11B	109.5
C2—C3—H3B	108.3	H11A—C11—H11B	109.5
H3A—C3—H3B	107.4	C10—C11—H11C	109.5
C3—C4—H4A	109.5	H11A—C11—H11C	109.5
C3—C4—H4B	109.5	H11B—C11—H11C	109.5
H4A—C4—H4B	109.5	N2—C12—C13	112.1 (2)
C3—C4—H4C	109.5	N2—C12—H12A	109.2
H4A—C4—H4C	109.5	C13—C12—H12A	109.2
H4B—C4—H4C	109.5	N2—C12—H12B	109.2
N10—C80—C30	112.8 (3)	C13—C12—H12B	109.2
N10—C80—H80A	109.0	H12A—C12—H12B	107.9
C30—C80—H80A	109.0	C12—C13—C14	110.9 (2)
N10—C80—H80B	109.0	C12—C13—H13A	109.5
C30—C80—H80B	109.0	C14—C13—H13A	109.5
H80A—C80—H80B	107.8	C12—C13—H13B	109.5
C40—C30—C80	119.5 (5)	C14—C13—H13B	109.5
C40—C30—H30A	107.4	H13A—C13—H13B	108.1
C80—C30—H30A	107.4	C13—C14—H14A	109.5
C40—C30—H30B	107.4	C13—C14—H14B	109.5
C80—C30—H30B	107.4	H14A—C14—H14B	109.5
H30A—C30—H30B	107.0	C13—C14—H14C	109.5
C30—C40—H40A	109.5	H14A—C14—H14C	109.5
C30—C40—H40B	109.5	H14B—C14—H14C	109.5
H40A—C40—H40B	109.5	N3—C15—C16	123.3 (2)
C30—C40—H40C	109.5	N3—C15—H15	118.4
H40A—C40—H40C	109.5	C16—C15—H15	118.4
H40B—C40—H40C	109.5	C17—C16—C15	118.9 (2)
C1—N1—C2	123.5 (2)	C17—C16—H16	120.6
C1—N1—C5	121.2 (3)	C15—C16—H16	120.6
C2—N1—C5	113.4 (3)	C16—C17—C18	118.5 (2)
N1—C5—C6	109.6 (5)	C16—C17—H17	120.7
N1—C5—H5A	109.8	C18—C17—H17	120.7
C6—C5—H5A	109.8	C19—C18—C17	118.9 (2)
N1—C5—H5B	109.8	C19—C18—H18	120.6
C6—C5—H5B	109.8	C17—C18—H18	120.6
H5A—C5—H5B	108.2	N3—C19—C18	122.7 (2)
C7—C6—C5	109.8 (5)	N3—C19—C20	117.2 (2)
C7—C6—H6A	109.7	C18—C19—C20	120.1 (2)
C5—C6—H6A	109.7	N4—C20—C19	119.5 (2)
C7—C6—H6B	109.7	N4—C20—H20	120.3
C5—C6—H6B	109.7	C19—C20—H20	120.3
H6A—C6—H6B	108.2		
			
N3—Cd—S1—C1	69.94 (10)	S1—C1—N1—C2	0.0 (4)
N4—Cd—S1—C1	127.66 (9)	N10—C1—N1—C5	0(100)
S3—Cd—S1—C1	−155.19 (8)	S2—C1—N1—C5	−17.3 (5)
S4—Cd—S1—C1	−73.14 (8)	S1—C1—N1—C5	162.8 (4)
S2—Cd—S1—C1	0.95 (8)	C3—C2—N1—C1	−120.0 (3)
N3—Cd—S2—C1	−131.74 (9)	C3—C2—N1—C5	76.1 (4)
S1—Cd—S2—C1	−0.97 (8)	C1—N1—C5—C6	88.3 (5)
N4—Cd—S2—C1	−72.54 (9)	C2—N1—C5—C6	−107.3 (4)
S3—Cd—S2—C1	96.17 (9)	N1—C5—C6—C7	−173.3 (4)
S4—Cd—S2—C1	133.80 (8)	N1—C1—N10—C80	0(40)
N3—Cd—S3—C8	−87.89 (9)	S2—C1—N10—C80	179.9 (2)
S1—Cd—S3—C8	129.01 (8)	S1—C1—N10—C80	0.0 (4)
N4—Cd—S3—C8	−148.37 (9)	N1—C1—N10—C50	0(100)
S4—Cd—S3—C8	0.51 (8)	S2—C1—N10—C50	13.8 (5)
S2—Cd—S3—C8	41.31 (10)	S1—C1—N10—C50	−166.1 (4)
N3—Cd—S4—C8	105.86 (9)	C30—C80—N10—C1	−74.1 (4)
S1—Cd—S4—C8	−103.38 (8)	C30—C80—N10—C50	92.0 (5)
N4—Cd—S4—C8	46.11 (10)	C1—N10—C50—C60	−95.8 (5)
S3—Cd—S4—C8	−0.51 (8)	C80—N10—C50—C60	97.3 (5)
S2—Cd—S4—C8	−166.33 (8)	N10—C50—C60—C70	179.0 (5)
S1—Cd—N3—C15	−110.58 (17)	C9—N2—C8—S4	179.07 (19)
N4—Cd—N3—C15	−178.32 (19)	C12—N2—C8—S4	1.9 (3)
S3—Cd—N3—C15	112.22 (17)	C9—N2—C8—S3	−0.9 (3)
S4—Cd—N3—C15	43.08 (17)	C12—N2—C8—S3	−178.15 (18)
S2—Cd—N3—C15	−50.92 (17)	Cd—S4—C8—N2	−179.2 (2)
S1—Cd—N3—C19	63.49 (18)	Cd—S4—C8—S3	0.82 (13)
N4—Cd—N3—C19	−4.25 (16)	Cd—S3—C8—N2	179.2 (2)
S3—Cd—N3—C19	−73.71 (17)	Cd—S3—C8—S4	−0.82 (13)
S4—Cd—N3—C19	−142.85 (16)	C8—N2—C9—C10	90.9 (3)
S2—Cd—N3—C19	123.15 (17)	C12—N2—C9—C10	−91.7 (3)
N3—Cd—N4—C20	1.00 (15)	N2—C9—C10—C11	179.9 (2)
S1—Cd—N4—C20	−130.34 (16)	C8—N2—C12—C13	88.1 (3)
S3—Cd—N4—C20	115.21 (16)	C9—N2—C12—C13	−89.3 (3)
S4—Cd—N4—C20	71.62 (17)	N2—C12—C13—C14	−172.8 (2)
S2—Cd—N4—C20	−69.06 (17)	C19—N3—C15—C16	−1.0 (3)
N3—Cd—N4—N4^i^	−174.1 (3)	Cd—N3—C15—C16	173.34 (18)
S1—Cd—N4—N4^i^	54.6 (3)	N3—C15—C16—C17	−0.1 (4)
S3—Cd—N4—N4^i^	−59.9 (2)	C15—C16—C17—C18	1.1 (4)
S4—Cd—N4—N4^i^	−103.4 (2)	C16—C17—C18—C19	−0.9 (4)
S2—Cd—N4—N4^i^	115.9 (2)	C15—N3—C19—C18	1.1 (3)
Cd—S2—C1—N10	−178.4 (2)	Cd—N3—C19—C18	−173.05 (17)
Cd—S2—C1—N1	−178.4 (2)	C15—N3—C19—C20	−178.9 (2)
Cd—S2—C1—S1	1.54 (13)	Cd—N3—C19—C20	6.9 (3)
Cd—S1—C1—N10	178.3 (2)	C17—C18—C19—N3	−0.2 (3)
Cd—S1—C1—N1	178.3 (2)	C17—C18—C19—C20	179.8 (2)
Cd—S1—C1—S2	−1.60 (13)	N4^i^—N4—C20—C19	178.0 (2)
N1—C2—C3—C4	55.2 (4)	Cd—N4—C20—C19	1.9 (3)
N10—C80—C30—C40	−47.3 (7)	N3—C19—C20—N4	−5.7 (3)
S2—C1—N1—C2	179.9 (2)	C18—C19—C20—N4	174.3 (2)

Symmetry codes: (i) −*x*, −*y*+1, −*z*+1.

### Hydrogen-bond geometry (Å, °)

**Table:**

*D*—H···*A*	*D*—H	H···*A*	*D*···*A*	*D*—H···*A*
C9—H9B···S4^ii^	0.99	2.83	3.815 (3)	171
C16—H16···S3^iii^	0.95	2.82	3.674 (3)	150
C3—H3B···Cg^iv^	0.95	2.99	3.853 (5)	147

Symmetry codes: (ii) −*x*, *y*+1/2, −*z*+3/2; (iii) *x*+1, *y*, *z*; (iv) *x*, *y*−1, *z*.
